# Nécrose et infection locales compliquant une envenimation par *Echis ocellatus* chez un enfant au Mali

**DOI:** 10.48327/mtsi.v3i1.2023.345

**Published:** 2023-03-20

**Authors:** Moussa Keita, Sanou Khô Coulibaly, Sanra Déborah Sanogo

**Affiliations:** 1 Clinique privée « Néné », Kati, Mali; 2 Faculté de médecine et dodontostomatologie, Université des sciences techniques et des tech-nologies de Bamako (USTTB), BP 1805 Bamako, Mali

**Keywords:** Enfant, Envenimation, Test de coagulation sur tube sec, Sérum antivenimeux, *Echis ocellatus*, Kolokani, Kati, Mali, Afrique subsaharienne, Child, Envenomation, Whole blood coagulation test, Antivenom, *Echis ocellatus*, Kolokani, Kati, Mali, Sub-Saharan Africa

## Abstract

Un enfant de 4 ans, vivant avec ses parents à Kolokani, une localité située à une centaine de kilomètres de Bamako a été mordu au niveau de l'index gauche par un serpent de l'espèce *Echis ocellatus* (fonfoni en langue locale). Après 2 semaines de traitement traditionnel, des complications locales ont été observées. L'enfant a été admis à la clinique Néné de Kati (Mali) le 19 juillet 2022. Les signes observés avaient été corrélés au degré d'envenimation et le test de coagulation sur tube sec avait montré des troubles de la coagulation, ce qui avait justifié l'administration d'antivenin. En raison d'une nécrose de la totalité de l'index, une amputation a été réalisée, avec des suites simples. Les morsures de serpent nécessitent une prise en charge adéquate afin de prévenir des complications telles que la nécrose et l'infection du site de morsure. L'administration d'antivenin est nécessaire si les troubles de la coagulation persistent. Un traitement chirurgical et une antibiothérapie à large spectre peuvent améliorer le pronostic vital.

## Abstract (en)

A 4-year-old child living with his parents in Kolokani, a town about 100 km from Bamako, was bitten on the left index finger by a snake of the species *Echis ocellatus* (fonfoni in local language). After 2 weeks of traditional treatment, local complications were observed. The child was admitted to the Néné clinic in Kati (Mali) on 19 July 2022. The signs observed were correlated with the degree of envenomation and the whole blood coagulation test showed coagulation disorders, which justified the administration of antivenom. Necrosis of the whole index finger required amputation of the finger, which was not followed by any complications. Snakebites require proper management to prevent complications such as necrosis and infection of the bite site. Administration of antivenom is necessary if coagulation disorders persist. Surgical treatment and broad-spectrum antibiotic therapy may improve the prognosis.

## Introduction

L'envenimation par morsure de serpent est une urgence médico-chirurgicale avec une morbidité et une mortalité élevées dans de nombreuses régions du monde [3]. Elles constituent un problème de santé publique négligé, surtout dans les pays tropicaux et subtropicaux. Les complications hémorragiques et infectieuses des morsures sont associées au retard de la prise en charge entretenu par les croyances culturelles traditionnelles et aux difficultés financières d'accès aux soins appropriés [1].

Nous rapportons le cas d'un enfant ayant présenté une nécrose et une surinfection de l'index gauche survenues suite à une morsure de serpent.

## Cas Clinique

Un enfant de 4 ans vivait avec ses parents à Kolokani, une localité située à une centaine de kilomètres de Bamako.

C'est un milieu rural et le patient avait été reçu à la clinique Néné le 19 juillet 2022 à 14 h après une morsure de serpent de l'espèce *Echis ocellatus* (fonfoni en langue locale) au niveau de l'index gauche au cours de travaux champêtres avec ses parents.

Le serpent n'avait pas été retrouvé après la morsure, mais avait été bien identifié et décrit par les parents. Ces derniers avaient alors effectué des traitements traditionnels à base de décoctions de plantes non spécifiées et de poudre noire appliquée sur le point de la morsure avant son admission. Après 2 semaines de traitement traditionnel, des complications locales avaient été observées: un œdème de la paume de la main gauche noté grade 3 selon la graduation de Larréché *et al.* [4] suivi d'une nécrose noirâtre, purulente et gazeuse d'odeur fétide, et d'une pâleur cutanéomuqueuse et palmo-plantaire (Fig. 1 et 2). Les parents ont consulté à la clinique médicale Néné pour une meilleure prise en charge. La séquence et la date d'apparition des signes n'avaient pas pu être précisées par les parents.

**Figure 1:**
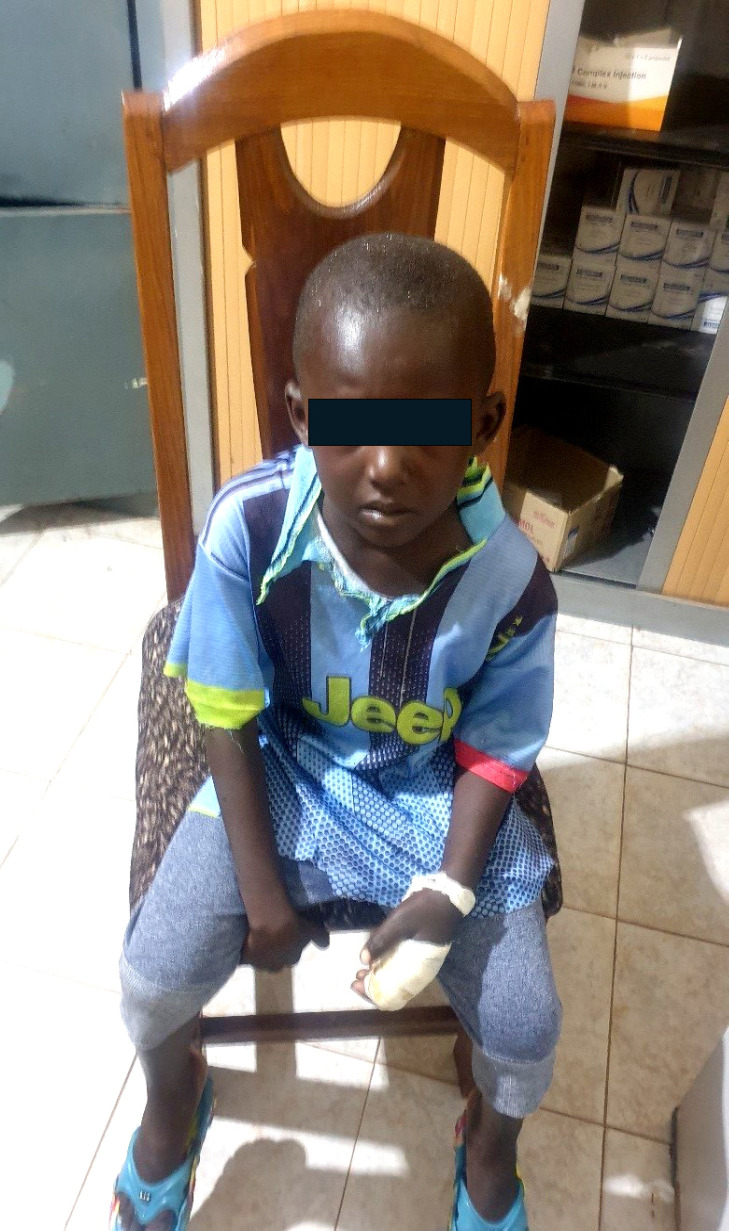
Arrivée du jeune patient deux semaines après la morsure Hospital presentation two weeks after snakebite

**Figure 1:**
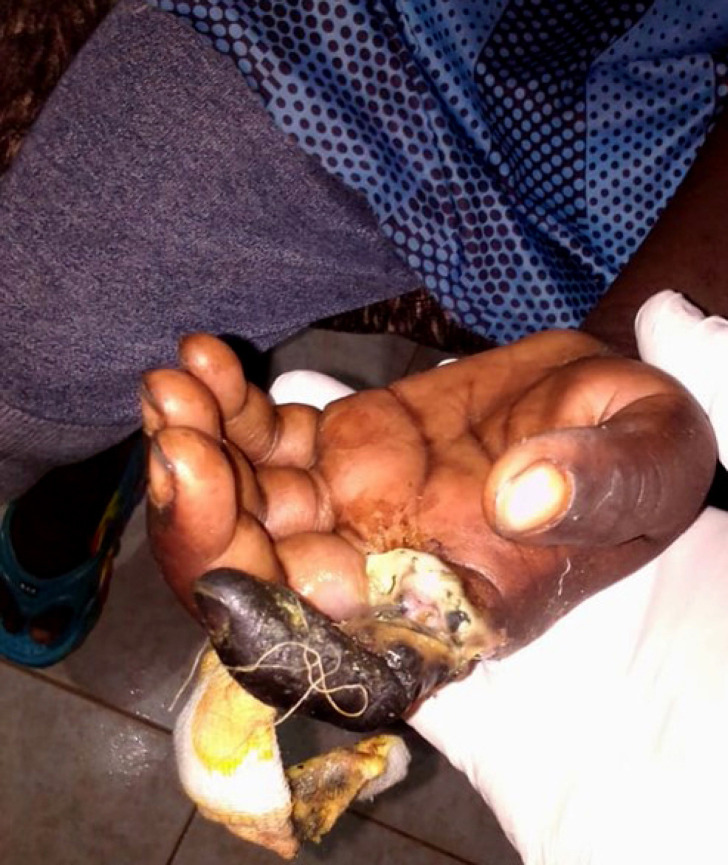
Nécrose de l'index gauche Left index finger necrosis

**Figure 3:**
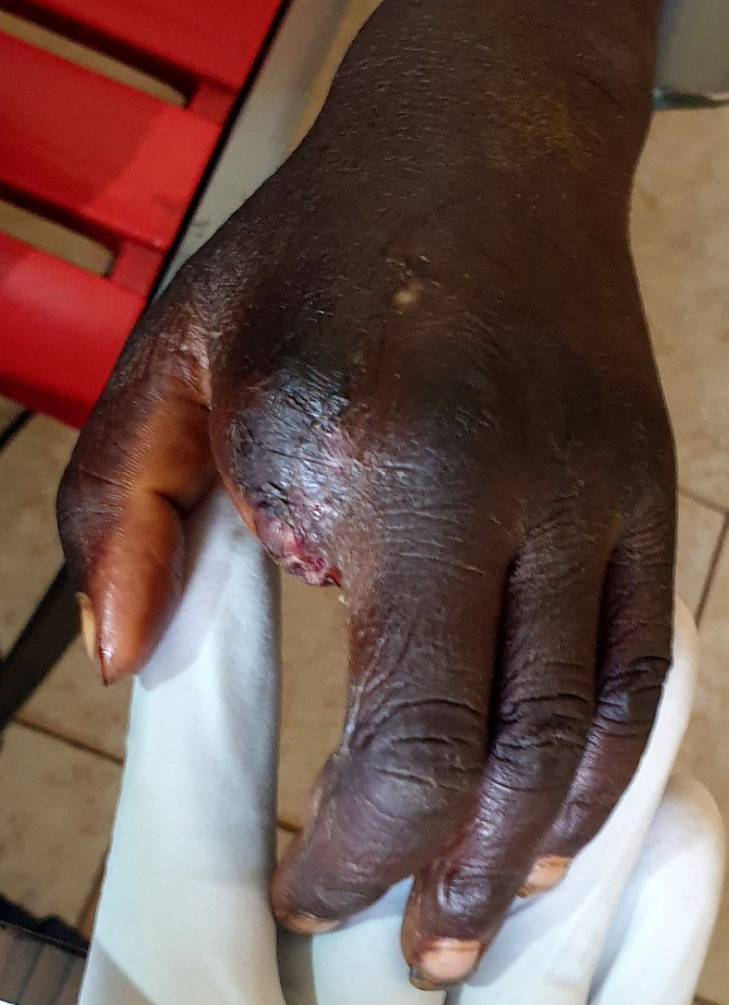
Cicatrisation après amputation de l'index Healing after amputation of index finger

**Figure 4:**
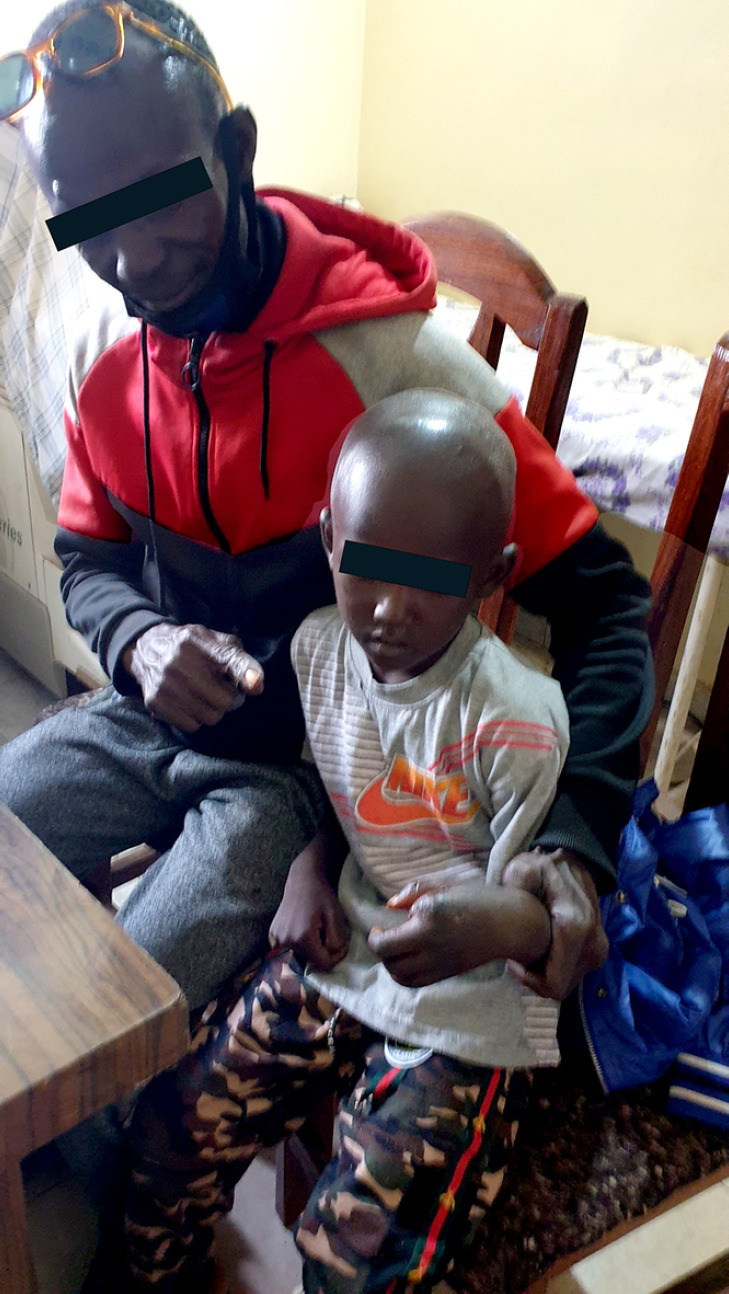
Guérison Recovery

L'examen clinique à l'admission avait objectivé une hyperthermie à 39 °C, un état général peu altéré, une anxiété et une agitation ainsi que la présence d'une gingivorragie. Le test de coagulation sur tube sec (TCTS) selon la méthode de Chippaux *et al.* [2] montrait des troubles de la coagulation (sang incoagulable); le bilan hématologique montrait un taux d'hémoglobine bas à 6 g/dl, un taux d'hématocrite de 18%, une hyperleucocytose supérieure à 21 G/L et une thrombopénie à 9 G/L, une glycémie et une créatininémie normales. Après le pansement de la plaie, nous avons procédé à une amputation de l'index gauche (Fig. 3). De l'antivenin (Inoserp™ Pan-Africa, Inosan Biopharma) à une dose de 20 ml dans 300 ml de sérum salé isotonique, une antibiothérapie à large spectre: amoxicilline et acide clavulanique (2 g par jour, en intraveineuse) ont été administrés. L'enfant a également reçu une transfusion de 2 poches de sang total iso-groupe iso-rhésus. Le deuxième TCTS réalisé 4 heures plus tard était normal, l’évolution était favorable après 7 jours d'hospitalisation malgré des séquelles fonctionnelles et esthétiques. Bien que complètement vacciné, l'enfant a reçu un sérum antitétanique comme préconisé par le protocole malien de prise en charge des morsures de serpent. L'enfant a continué à prendre du fer *per os* et une antibiothérapie de 14 jours après l'amputation. Le suivi post-hospitalier a confirmé l’évolution favorable et la parfaite cicatrisation (Fig. 4).

## Discussion

Au Mali, dans les régions ainsi que dans les cercles, les morsures de serpent sont particulièrement dangereuses du fait des difficultés d'accès aux centres de santé, du recours à la médecine traditionnelle, du manque de formation du personnel soignant et de la présence fréquente et abondante d’*Echis ocellatus.* Cette dernière est une vipère agressive et très dangereuse, responsable de manifestations cliniques hémorragiques et de complications nécrotiques. Ce cas clinique montrait un syndrome vipérin hémorragique, dû à un trouble de la coagulation, et une nécrose humide purulente, douloureuse, un œdème limité au niveau de la paume. Aucune des différentes techniques de traitement traditionnel n'a fait la preuve de son efficacité. Les traitements traditionnels (décoctions des plantes variables, talismans, garrot, pierre ou poudre noire) sont responsables du retard de consultation (2 semaines dans notre cas), ce qui met en péril la vie du patient. La décoction de plantes, très utilisée dans toute l'Afrique, n'apporte aucun bénéfice et peut être délétère en raison de la toxicité de certaines plantes et de dosages imprécis [1, 3].

L'immunothérapie antivenimeuse reste le seul traitement efficace contre les envenimations ophidiennes. Ces indications sont actuellement bien claires [1]. Elle doit être utilisée pour toute envenimation caractérisée par des signes patents, locaux (œdème, nécrose, saignement) ou systémiques (syndrome hémorragique, troubles neurologiques). Les complications observées des morsures sont dues, notamment, à l'effet direct des enzymes du venin, à l'altération de la microcirculation locale, à la colonisation bactérienne de différentes origines (gueule du serpent, peau du patient, environnement) et surtout à l'utilisation des techniques traditionnelles. Les signes observés avaient été corrélés au degré d'envenimation, et le TCTS avait montré des troubles de la coagulation, ce qui avait justifié l'administration d'antivenin à plus de 14 jours après la morsure [1, 2]. Le traitement antivenimeux que nous avons utilisé était l'Inoserp^TM^ Pan-Africa composé de fragments d'immunoglobulines hautement purifiées, indiqué pour le traitement des patients présentant des signes cliniques d'envenimation par serpent (en particulier, *Naja nigricollis, Dendroaspis polylepis, Echis ocellatus* et *Bitis arietans)*, qui s'est avéré simple d'utilisation et efficace. Il est le plus utilisé au Mali. La posologie dépend d'une part, de la quantité de venin injectée par le serpent, c'est-à-dire de l'intensité des symptômes présentés par le patient, et d'autre part, de la capacité de neutralisation de l'antivenin vis-à-vis des espèces locales [3]. Ce cas clinique confirme l'intérêt de certains tests, notamment le TCTS pour le diagnostic et la surveillance du syndrome hémorragique [3].

## Conclusion

Les morsures de serpent nécessitent une prise en charge adéquate afin de prévenir des complications telles que la nécrose et l'infection du site de morsure. L'administration du sérum antivenimeux est nécessaire si les troubles de la coagulation persistent. Un traitement chirurgical et une antibiothérapie à large spectre peuvent améliorer le pronostic vital.

## Contributions Des Auteurs

Tous les auteurs ont participé à la conception, à la relecture et à la validation du manuscrit.

## Liens D'intérêts

Les auteurs ne déclarent aucun conflit d'intérêts.
